# Translation and First Pilot Validation Study of the “Undergraduate Nursing Student Academic Satisfaction Scale” Questionnaire to the Spanish Context

**DOI:** 10.3390/ijerph18020423

**Published:** 2021-01-07

**Authors:** María Dolores Guerra-Martín, Alejandro Cano-Orihuela, Raúl Martos-García, José Antonio Ponce-Blandón

**Affiliations:** 1 Department of Nursing, University of Seville, 41009 Seville, Spain; 2 University Hospital Son Espases, 07120 Palma de Mallorca, Spain; alejandrocanoorihuela@gmail.com; 3 Red Cross Nursing School, University of Seville, 41009 Seville, Spain; rmartos@cruzroja.es

**Keywords:** personal satisfaction, students, nursing, surveys and questionnaires, validation studies

## Abstract

Satisfaction helps nursing students to develop skills and improve their academic performance, hence the importance of assessing it by means of a reliable instrument. The objective was to translate and culturally adapt the “Undergraduate Nursing Student Academic Satisfaction Scale” (UNSASS) instrument to the Spanish context. A cross-sectional study was conducted with a representative sample of 354 fourth-year nursing students from University of Seville, Seville, Spain. The validation process was carried out in five phases as follows: direct translation, synthesis of the translations, back translation, consolidation by a panel of experts, and pilot test with nursing students. After two rounds among two expert committees, the Content Validity Index (CVI) varied from 0.85 to 1, obtaining a CVI above 0.8 with the global questionnaire. A scale composed of 48 items and 4 subscales was obtained, resulting in a Cronbach’s α coefficient of 0.96. Within the subscales, this coefficient varied between 0.92 and 0.94. No statistically significant differences were found between the total satisfaction of the scale and gender and teaching unit. An inversely proportional relationship was found between the age and the “Support & Resources” scale. The “Escala de Satisfacción Académica del Estudiante de Enfermería” (ESAEE) scale was obtained, translated, and adapted to the Spanish context from the UNSASS scale, with satisfactory consistency and validity.

## 1. Introduction

The new focus of universities is to become facilitating centers for teaching, where the students are able to acquire knowledge through friendly methods and where the institutions base their efforts on promoting a healthy environment of well-being [1,2].

Critical and creative thinking is demanded from health professionals, and nursing education has evolved to adapt to these new requirements. This process has gradually taken place thanks to efficient professional competences aimed at obtaining a high level of satisfaction for the user of the educational system [3].

Students’ satisfaction helps build self-confidence and is a source of support to develop skills and acquire knowledge, thus helping to improve academic performance [4,5].

The current generation of students is flooded with a great variety of stimuli, with an element that reinforces the motivation to learn in the students being important; in this sense, satisfaction is very much connected to the increase in motivation, through which they learn more and better, which is useful for their future professional practice [6]. However, satisfaction in relation to obtaining any degree is complemented by stressors such as suffering, diseases, disabilities, or death of patients [7].

The non-intellective competences involved in academic performance can be important resources for promoting academic adjustment and satisfaction, favoring the retention and persistence processes in college. In the same way, working on the non-cognitive skills closely linked with study satisfaction could also improve performance and academic success, thereby increasing domain-specific satisfaction. In this case, for example, individual work on intrinsic motivation or group work that enhances the ability to relate to one’s fellow students could improve satisfaction with one’s study habits [8].

Furthermore, some studies support the finding that the environment at university can be perceived as stressful and that developing the ability to handle negative situations effectively increases perceived support from peer relationships and can create a virtuous cycle that helps students improve this competence [9]. Thus, some authors suggest the importance of developing one’s own scale to measure the satisfaction of Nursing Degree students [7], carrying out experiences in which it is clear that these students complement the stressors that young people commonly have with those additional derived from the practical environment, in which day by day they find stress-generating stimuli. Moreover, there is the need that these students have to establish relationships with other health professionals, and the fact of having to play a new role for which they have not yet been fully trained.

Thus, there are various studies whose objective was to validate standardized scales targeted at evaluating nursing students’ satisfaction, by dealing with either one or more of its aspects or factors, and directed towards a specific idiomatic and/or cultural context. Currently, there are validated scales that evaluate satisfaction and effectiveness in the clinical learning setting, as well as satisfaction in clinical practices [10,11,12,13]. In the case of Asadizaker et al. [4], with their “Satisfaction with First Clinical Practical Education” (SFCPE) scale, they evaluated in a more comprehensive manner this realm of nursing education, through the “Instructor performance” (α = 0.92), “Integrated plan” (α = 0.82)”, “Feelings and perceptions” (α = 0.78), “Learning atmosphere” (α = 0.73), “Scheduling” (α = 0.70), “Facilities” (α = 0,65), and “Access to professionals” (α = 0.60) subscales. Furthermore, given the importance that the first experience in a clinical setting, the main factor assessed by this scale, is the cornerstone of the future in professional nursing for all nursing students, the authors defend the use of this instrument, concluding that it is valid and reliable.

In the scope of the European Space of Higher Education, Lepiani et al. [14] conducted a study focused on the satisfaction of first-year nursing students when addressing Basic knowledge, the Organization of teaching, the Skills developed, the Teaching–learning process, Access and attention in the students, and the Curriculum and its structure as study factors. Satisfaction assessment scales have also been described in the university context, not only in the nursing field, which have shown to be useful and could also be applied in contexts of assessment of the satisfaction of undergraduate nursing students [15].

In contrast, among the scales of multidimensional appreciation are studies such as the one focused on determining nursing students’ satisfaction [16], where a level of satisfaction in 11 factors was determined. Additionally, the study developed by Gloria and Ortiz [1] established the quality of life level and the factors related to it in such students, when applying the “Calidad de Vida y Satisfacción” (Q-LES-Q) questionnaire.

Despite the usefulness of all these instruments, a multidimensional, validated, and reliable “Undergraduate Nursing Student Academic Satisfaction Scale” (UNSASS) was found focusing on the measurement of satisfaction of nursing students with a Nursing Degree and all the integral aspects that make up the student’s learning period. This scale was developed by Dennison and El-Masri [17] and validated in the Canadian context, whose function is to evaluate the level of satisfaction over four dimensions, namely “In-Class Teaching”, “Clinical Teaching”, “The Program”, and “Support & Resources”, where each scale obtained Cronbach’s alpha coefficients of 0.92, 0.91, 0.91, and 0.74, respectively, and of 0.96 for the entire scale, which denoted an excellent overall internal consistency.

It is true that other instruments have been developed to evaluate the satisfaction of nursing students, in different cultural contexts, such as the work of Chen and Lo [18], validating the “Nursing Student Satisfaction Scale” (NSSS) instrument for its use in the USA context with high internal consistency (α = 0.96), where satisfaction was evaluated in three factors, namely Curriculum and teaching, Environment, and Professional social interaction. Subsequently, Domingues et al. [19] once again validated this tool, the NSSS scale, although in this case for the Brazilian context. It was composed of three factors, namely “Curricular dimension and teaching”, “Environmental dimension”, and “Social/Professional interactions”. Despite the recognition of the usefulness of this scale in different contexts, it clearly covers a smaller set of scenarios in its studied dimensions, excluding aspects as relevant as the support and necessary resources and the differentiation between clinical learning environments and academic environments (“in-class” learning). These aspects are however effectively addressed by the UNSASS scale, proving to be one of the most complete due to its properties of internal consistency, reliability, measurement of errors, construct validity, structural validity, and criterion validity. Furthermore, the scale follows the standardized quality assessment criteria methodology of studies on measurement properties of health status measurement instruments [20].

Thus, a translation and validation research of the aforementioned scale to the Spanish context were justified. We therefore proposed the research as this study’s objective to translate and culturally adapt the multidimensional, validated, and reliable “Undergraduate Nursing Student Academic Satisfaction Scale” instrument to the Spanish context. The study also aimed to verify the level of satisfaction of fourth-year nursing students and the existing relationships between the level of satisfaction and gender, age, or the teaching units to which they belonged.

## 2. Materials and Methods 

### 2.1. Design and Participants

A validation study of the “Undergraduate Nursing Student Academic Satisfaction Scale” (UNSASS) questionnaire adapted to the Spanish context was conducted, which included a non-experimental, quantitative, descriptive, and cross-sectional pilot test, between December 2018 and July 2019, where data collection took place in April 2019.

The study population were the 354 fourth-year nursing students belonging to the Virgen Macarena, Virgen del Rocío, or Virgen de Valme teaching units, from the School of Nursing, Physiotherapy and Podiatry of the University of Seville, Seville, Spain, where a stratified sampling was conducted respecting the proportion of students of the target population. The students were recruited by means of e-mail messages, and the instrument was provided online using the Google Forms tool. From the study population divided by teaching unit composed of 74 from Virgen del Rocío, 226 from Virgen Macarena, and 54 from Virgen de Valme, a final sample of 32 students (9.04%) of the entire school was obtained, of which 7 (9.46%), 19 (8.41%), and 6 (11.11%) were from each of the teaching units, respectively, numbers that fall within the 30–40 students estimated in the scientific literature [21].

In addition to asking about gender, age, and teaching unit, the instrument was applied to fourth-year students since they were immersed in the context of undergraduate nursing studies for a longer period of time and, therefore, possessed more knowledge, excluding those who did not understand Spanish and who did not attend the previous years in that school.

### 2.2. Translation and Cultural Adaptation Process

The instrument used for validation in the Spanish context was the “Undergraduate Nursing Student Academic Satisfaction Scale” (UNSASS) [17]. To this end, the guidelines for cultural adaptation and validation of health questionnaires by Ramada, Serra, and Declós [22] and by Romero-Martín, Gómez-Salgado, De la Fuente-Ginés, Macías-Seda, García-Díaz, and Ponce-Blandón [23] were followed, consisting of five phases, namely direct translation, synthesis, back translation, consolidation by expert committees, and pilot test of the questionnaire.

Initially, two independent and bilingual nurses with work experience and knowledge of the Spanish university system in force conducted a linguistic, cultural, and conceptual translation of the instrument [24]. Subsequently, the researchers synthesized both translated versions comparing by pairs, where the existing discrepancies between the two versions were identified and discussed, reaching consensus. The consensual version in Spanish was back-translated into English by two independent translators, who did not have access to the original version. Subsequently, pair corrections were made, and the prototype of the scale called “Escala de Satisfacción Académica del Estudiante de Enfermería (ESAEE)” was created. For logical appearance validity, two expert committees were created with a total of 13 participants—one was made up of 7 professors from diverse university settings and the other was constituted by 6 fourth-year nursing students [21]. The objective of both expert committees was to assess whether each item was understandable, with sufficient clarity in each of them.

### 2.3. Applicability and Feasibility

In the pilot phase, each of the participants was asked to complete the version of the ESAEE instrument translated and adapted to the Spanish context and, later on, they were given the choice to express if they found any difficulty in understanding any of the items, as well as to leave comments on them. In this sense, if at least 15% of the students had difficulties with any item, it should be reviewed; but none of the participants found any difficulty in answering the questions. We also asked them to indicate the time they needed to answer the survey, the result being slightly less than 15 min.

Likewise, it was considered that, in adaptations, it is relevant not only to show evidence of a possible linguistic equivalence between the original and the adapted instruments, but also to state that the adaptations are equivalent from a conceptual point of view [24].

When calculating the relevance or validity of the questionnaire, the “Content Validity Index” (CVI) was used, in which, for each item to be considered acceptable in the final questionnaire, its relevance had to be assessed with a value of 3 or 4 out of 4 on a Likert scale by more than 78% of the experts [25,26], that is, a CVI equal to or higher than 0.78, and that it was understandable for at least 80% of the experts [21,27].

Those items that did not meet the aforementioned requirements were reviewed and reassessed, contacts being made with the primary translators and with those in charge of the back translation, in order to verify that the changes we were going to implement faithfully reflected the original scale. With the translators’ approval, a series of changes were introduced in some items; the others were kept unchanged following the translators’ suggestions.

### 2.4. Statistical Analysis

When assessing the internal consistency or reliability of the questionnaire, its Cronbach’s alpha coefficient was calculated, where a value above 0.7 was considered acceptable, which reveals a strong relationship among the questions of the test, either in each dimension or subscale or in the entire questionnaire [28]. For the construct validity, a confirmatory factor analysis (CFA) was used, and a previous verification of the suitability was checked with the Bartlett sphericity test and the Kaiser–Meyer–Olkin (KMO) coefficient. A significance of *p* < 0.05 for the Bartlett test and a value of KMO > 0.60 were considered acceptable as recommended in the literature [29]. The extraction method used to perform the CFA was the principal component analysis, and the rotation method used was the Varimax method with Kaiser normalization.

When describing the gender, age, and teaching unit variables, the subscales and items of the questionnaire, means, standard deviations and percentages were used.

The Shapiro–Wilk test and the Levene’s test were used to observe normality distribution, as well as to determine whether there was homogeneity in the variances, in addition to parametric tests such as the Student’s *t*-test and the ANOVA test or non-parametric ones, like Kruskal-Wallis or Mann-Whitney U. For the relationship of two continuous quantitative variables, Pearson’s correlation test was performed.

In order to perform the statistical data analysis, the SPSS© statistical software, version 21.0 (IBM Corp., Armonk, NY, USA) was used.

### 2.5. Ethical Considerations

Prior to the translation and validation process, authorization was asked from the authors of the UNSASS questionnaire, and they gave permission to use the UNSASS scale for its adaptation and use in the present study [17].

The participants of both expert committees (professors and students) and the students who participated in the pilot test were previously informed about the purpose of the study and they provided informed voluntary written consent. Their information was registered anonymously so it would not be possible to identify participants´ answers, guaranteeing anonymity and data confidentiality at all times. The study was approved by the Research Ethics Committee from the Spanish Red Cross Nursing College, University of Seville, with reference number 10/2018. According to this report, the study meets the requirements for research with human beings and complies with current regulations in Spain and the European Union regarding research issues.

## 3. Results

### 3.1. Translation and Adaptation Phase

After the described translation process and assessment, the initial version of the instrument, after going through the expert panel, can be found as supplementary information to this article (Table S1, Supplementary Materials), with its variables (type and operational definition) summarized in Table 1.

### 3.2. Item Creation Process and Content Validity

When presenting a CVI above 0.78 or when being considered clear by at least 80% of the experts in both the first and second rounds, the reevaluated items were accepted as relevant and were included in the final version of the scale.

In the first round of item appreciation by the expert committees, seven items (3, 15, 28, 30, 31, 42, and 44) did not reach the clarity appreciation cutoff point in at least 80% of the experts, and there were also six items (4, 15, 28, 31, 44, and 45) with a CVI below 0.78. Those items were subjected to a second evaluation round with the improvements proposed by the experts and a reassessment of the translation process. In any case, in this first round of the expert panel of professors, some of the suggestions for improvement that the experts proposed for each of the items that did not meet the validity or relevance criteria were taken into account.

Unlike in the panel of professors, the panel of students did not find objections in the items when assessing their clarity and relevance.

Therefore, the items in which professors had discrepancies were reevaluated, rechecking each of the steps previously performed, again comparing the previous versions of the scale, and contacting the primary and native translators who carried out the translations, to verify that the modifications that we were going to make could be a reflection of the original scale, in order to maintain fidelity in the process, since it was not possible to move away from its essence. With the approval of the translators, some modifications were introduced in the items, and in others, they were kept the same following the suggestions of the translators.

Once the items had been modified, a second analysis was made by the panel of professors in order to verify that the changes made were the ideal ones to achieve optimal clarity and relevance of each of the items. In this second round of the validation process, all the reevaluated items obtained a CVI above 0.78 (Table 2), the reason why they were accepted as relevant and were included in the final version of the scale. They observed that, in its integrity, the scale was expressed with sufficient clarity, thereby concluding this phase of the validation process, including all items for the final version of the scale, even the reassessed items.

### 3.3. Results of the Pilot Study

The ESAEE scale was administered to a sample of 32 students. A total of 78.1% of participants (*n* = 25) were women. The mean age of the sample was 22.2 years (21–27), SD = 1.62). The distribution of participants among the different teaching units was 21.8% (*n* = 7), 59.3% (*n* = 19), and 18.7% (*n* = 6) for “Virgen del Rocío”, “Virgen Macarena”, and “Virgen de Valme” units, respectively.

Table 3 shows the descriptive of the score obtained (mean, standard deviation, and the percentage of the mean score related to the maximum value of each of the subscales and the ESAEE scale as a whole). The subscale 4 “Support & Resources” was the best valued subscale by nursing students. No comprehension or legibility problems were identified for any of the items. 

Table 4 presents the mean and standard deviation for each of the items and for each of the subscales.

### 3.4. Internal Consistency and Reliability

The four subscales that constitute the ESAEE scale obtained Cronbach’s alpha coefficients of 0.94 (In-Class Teaching subscale), 0.94 (Clinical Teaching subscale), 0.92 (Program Design and Delivery subscale), and 0.92 (Support & Resources subscale). The ESAEE scale obtained the highest Cronbach’s alpha coefficient, i.e., 0.96.

### 3.5. Internal Validity of the Scale

Regarding the confirmatory factor analysis (CFA), before the CFA, KMO = 0.93 and *p* < 0.001 was achieved in the Bartlett test. The CFA results revealed and confirmed the four original factors that accounted for 67.3% of the total variance and a factor loading above 0.4 in all the items. Table 5 presents, through the rotated component matrix, the values of the CFA model and the standardized coefficients of the model items that best fit (only factor loading values above 0.4 are expressed).

### 3.6. Hypothesis Contrast Analysis

The relationship of gender and teaching unit with the satisfaction of the surveyed students was analyzed, not only in each of the factors or dimensions but also in the entire scale, and no relationship was observed. However, in the case of the relationship between age and satisfaction there was no relation between them, both in the entire scale and in all the subscales, except for the “Support & Resources” subscale (*p* = 0.003; Pearson’s correlation coefficient of −0.513), which indicated an inversely proportional relationship, with moderate intensity.

## 4. Discussion

The final version of the ESAEE scale is composed of 48 items divided into 4 subscales (In-Class Teaching, Clinical Teaching, Program Design and Delivery, and Support & Resources), identically to the original UNSASS scale [17]. All the items, as Dennison and El-Masri [17] did in their development of the UNSASS scale, were evaluated both in terms of clarity and of relevance, this last characteristic by means of the CVI. Similarly, in order to maintain fidelity with the original in the adaptation of the instrument, the same Likert-type scale scoring was followed, and no strata were proposed for the classification of satisfaction.

By means of the expert committees (professors and students), it was verified that the ESAEE scale presents logical appearance validity. The reliability of the UNSASS scale was assessed using Cronbach’s alpha, and showed excellent reliability for the entire scale and for the subscales, with the ESAEE scale presenting reliability levels even higher than the original. However, it is necessary to highlight that the calculation of Cronbach’s alpha was not performed from the perspective of a scale made up of 5-point ordinal variables, as suggested by some in the literature [30,31] but rather as nominal polycotomic variables. However, in any case, this same literature suggests that with this type of analysis the Cronbach’s alpha would probably have been even higher.

With respect to the “Satisfaction with First Clinical Practical Education” (SFCPE) questionnaire developed by Asadizaker et al. [4] to assess satisfaction in the nursing students with their first clinical setting, it consists of seven factors, and there are similarities when analyzing the nursing student’s satisfaction between the two scales, although the ESAEE scale is more compact for having fewer factors, four specifically.

In the first subscale of ESAEE, mostly devoted to theoretical teaching in classrooms, there is no correlation with the SFCPE scale, since the latter is more focused on the clinical scope. However, the second subscale, “Clinical Teaching”, is in fact widely adopted and, at the same time, subdivided into several factors that can relate between the subscales. In this sense, the SFCPE adopts factors like “Feelings and perceptions” (Factor 3) (related to items 18, 19, and 29 from ESAEE), “Clinical atmosphere” (Factor 4) (related to items 21, 23, and 25 from ESAEE), “Instructor performance” (Factor 1) (related to items 22, 24, 26, 30, and 31 from ESAEE), or “Access to professionals” (Factor 7) (related to items 17, 20, and 27 from ESAEE). The third subscale in ESAEE, “Program Design and Delivery”, is related to the SFCPE scale in the following factors: 2. “Integrated plan” and 5. “Scheduling”. Finally, the “Support & Resources” subscale from ESAEE is related to Factor 6. “Facilities” of the SFCPE scale. 

In relation to gender, no statistically significant difference was found between satisfaction and this variable. This coincides with the study by Salamonson et al. [32], in which no relationship was found between satisfaction and the age of the nursing students, coinciding with the results obtained in the ESAEE scale, with the exception of the “Support & Resources” subscale, where the relationship with satisfaction was in fact statistically significant. Studies such as the ones by Milton-Wildey et al. [33] and Domingues et al. [19] set forth that younger students present greater satisfaction levels than older students, which is in agreement with the results obtained in the “Support & Resources” subscale from ESAEE.

In the ESAEE scale, total satisfaction was high (75%). Regarding the decreasing order of the students’ satisfaction levels with the different subscales, we found that the “Support & Resources” subscale (83.8%) was the best valued, followed by “Clinical Teaching” (78.4%), “Program Design and Delivery” (72.6%), and, in fourth and last place, “In-Class Teaching” (70.8%).

The “In-Class Teaching” subscale from ESAEE obtained a higher result than that obtained in the “Cuestionario de Satisfacción del Estudiante” by Jiménez et al. [34], where the “Desempeño del profesor” factor (related to the “In-Class Teaching” subscale from ESAEE), was in second place, but with a percentage of 64%. The results obtained in our study (mean of 3.54) are in agreement with those obtained by Domingues et al. [19] with a mean of 3.57 for the “Curricular dimension and teaching” factor.

The results obtained for the “Clinical Teaching” subscale (78.4%) from ESAEE were consistent with those obtained in the study by Espeland and Indrehus [10], where 70% of the students were satisfied with the “Clinical practice”.

The “Program Design and Delivery” from ESAEE is related in the study by Lepiani et al. [18] with the “Organization of teaching” factor, which obtained a mean score of 3.81 in the students’ satisfaction levels, similarly to our study (3.63). In the study by Domingues et al. [19], this subscale is related to the “Curricular dimension and teaching” factor, which obtained a mean score of 3.57, also similar to the result obtained in the “Program Design and Delivery” subscale.

The results of the “Support & Resources” subscale from ESAEE, with items related to the infrastructures and to the administration and services staff, are in agreement with those obtained in the study by Pecina [3], where the areas best evaluated were “IT services” and “Infrastructures”. In the study by Lepiani et al. [14], the “Facilities” factor was the worst evaluated (mean of 2.87); however, this result does not coincide with the higher satisfaction level obtained in subscale 4 (mean of 4.19).

A very high reliability was obtained in the ESAEE scale (α: 0.96), coinciding with the values obtained in the studies by Asadizaker et al. [4] and Baykal et al. [14]. In addition, Domingues et al. [19] and Dennison and El-Masri [17] reached Cronbach’s α coefficients of 0.92, 0.97, 0.93, and 0.96, respectively; however, lower results, though reliable, were obtained in Pecina [3] and in Salamonson et al. [29], namely 0.83 and 0.80, respectively.

As a limitation of this research, we can mention having conducted an exploratory study of the ESAEE questionnaire, without accompanying it with a reliability test (test and re-test), in addition to only selecting students from the last year of the nursing program. This would have allowed us to calculate the interclass correlation coefficients, which would have given much more consistency to the study. Thus, as an improvement proposal, we shall conduct a reliability test, by having the participants complete the ESAEE questionnaire a second time 15 days after its first application, in addition to expanding the selection to students from the third year, who also have clinical practices as well as theoretical classes. Another limitation to highlight is the absence of an external evidence of validity, given that, due to the scarcity of specific scales available of this type, it would also be very difficult to identify a “gold standard”. As it is a first pilot validation study of the scale and, therefore, a line of research in which the authors continue to investigate, the inclusion of a comparison with an external evidence of validity could be suggested for the future.

## 5. Conclusions

Obtaining the satisfaction level of undergraduate nursing students represents an opportunity to know the aspects that can be improved, in order to implement measures leading to better quality in the studies, from the point of view not only of the curricula, but also of their transposition to theoretical classes, care practices, and the organization of the Nursing School. The aforementioned will improve how the students cope with their entry in the near future into the nursing profession and, with that, into the professional health care provided to people. This research could also be useful for a future review of the aforementioned aspects, either by the Nursing Schools or by the universities.

The Escala de Satisfacción Académica del Estudiante de Enfermería (ESAEE) scale was developed through a 5-phase validation process, adapted to the Spanish context. It consists of 48 items encompassed in four appreciation dimensions (In-Class Teaching, Clinical Teaching, Program Design and Delivery, and Support & Resources), with a Content Validity Index and a Cronbach’s α sufficiently high and similar to the original version of the validated questionnaire (UNSASS), which signifies sufficiently high internal consistency and validity of the content of the questionnaire for its validation. Only one moderate negative correlation was observed between the “Support & Resources” subscale and age.

For the future, we intend to conduct a multicenter research study with Nursing Schools from other universities, with a larger sample of nursing students and from different years, in addition to conducting not only a quantitative but also a qualitative approach, since in the world of perceptions and feelings, as in the case of the study of satisfaction, it is necessary to know more in depth what those students feel.

## Figures and Tables

**Table 1:** Variables, types, and operational definitions.

Variable	Type	Operational Definition
Age	Discreet and quantitative	Years old
Gender	Dichotomous, nominal, and qualitative	Man, Woman
Teaching Unit	Nominal and qualitative	Virgen del Rocío, Virgen de Valme, Virgen Macarena
Satisfaction (Scale total)	Quantitative	48–240
Satisfaction (In-Class Teaching subscale)	Quantitative	16–80
Satisfaction (Clinical Teaching subscale)	Quantitative	15–75
Satisfaction (Program Design and Delivery subscale)	Quantitative	12–60
Satisfaction (Support & Resources subscale)	Quantitative	5–25

**Table 2:** Content Validity Index (CVI) of the reevaluated items after the last round with the expert committees.

No.	Item	Content Validity Index
4	Faculty members make an effort to understand difficulties I might be having with my course work	0.85
15	Faculty members create a good overall impression	0.85
28	Clinical instructors provide enough opportunities for independent practice in the lab and clinical sites	1
31	Faculty members behave professionally	0.85
44	The secretaries are caring and helpful	0.85
45	The secretaries behave professionally	1

CVI: Content Validity Index.

**Table 3:** Mean, standard deviation, and percentage of the mean score in relation to the possible maximum of each of the subscales and of the entire “Escala de Satisfacción Académica del Estudiante de Enfermería” (ESAEE) scale.

Descriptive Parameter	In-Class Teaching Subscale	Clinical Teaching Subscale	Program Design and Delivery Subscale	Support & Resources Subscale	Total Satisfaction
Mean	3.54	3.92	3.63	4.19	3.75
Standard Deviation	0.22	0.19	0.27	0.08	0.30
Percentages	70.8%	78.4%	72.6%	83.8%	75.0%

**Table 4:** Mean values and standard deviation for items, subscales, and total ESAEE scale.

Items	Mean	SD	Items	Mean	SD
**“In-Class Teaching” subscale**	3.54	0.22	**“Clinical Teaching” subscale**	3.92	0.19
1. I can freely express my academic and other concerns to faculty members	3.81	1.03	17. Clinical instructors are approachable and make students feel comfortable about asking questions	4.00	0.84
2. Faculty members are easily approachable	3.69	0.82	18. Clinical instructors provide feedback at appropriate times, and do not embarrass me in front of others (classmates, staff, patients and family members)	4.00	1.02
3. Faculty members make every effort to assist students when asked	3.72	0.81	19. Clinical instructors are open to discussions and difference in opinions	3.75	0.95
4. Faculty members make an effort to understand difficulties I might be having with my course work	3.44	0.98	20. Clinical instructors give me sufficient guidance before I perform technical skills	4.09	0.86
5. Faculty members are usually available after class and during office hours	3.75	0.80	21. Clinical instructors view my mistakes as part of my learning	4.00	1.05
6. I can freely express my academic and other concerns to the administration	3.56	0.95	22. Clinical instructors give me clear ideas of what is expected from me during a clinical rotation	3.78	1.07
7. Faculty are fair and unbiased in their treatment of individual students	3.66	0.90	23. Clinical instructors facilitate my ability to critically assess my client’s needs	3.72	1.08
8. Faculty members provide adequate feedback about students’ progress in a course	3.34	0.90	24. Clinical instructors assign me to patients that are appropriate for my level of competence	4.13	0.87
9. I receive detailed feedback from faculty members on my work and written assignments	3.19	1.15	25. Clinical instructors give me verbal and written feedback concerning my clinical experience	3.91	1.03
10. Channels for expressing students’ complaints are readily available	3.09	1.30	26. Clinical instructors demonstrate a high level of knowledge and clinical expertise	3.97	0.86
11. Faculty members are good role models and motivate me to do my best	3.59	0.80	27. Clinical instructors are available when needed	3.84	0.88
12. The administration shows concern for students as individuals	3.34	1.07	28. Clinical instructors provide enough opportunities for independent practice in the lab and clinical sites	3.81	1.00
13. Faculty members demonstrate a high level of knowledge in their subject area	3.69	0.78	29. Clinical instructors encourage me to link theory to practice	3.69	0.97
14. Faculty members take the time to listen/discuss issues that may impact my academic performance	3.44	1.05	30. Instructions are consistent among different clinical and lab instructors	3.78	0.97
15. Faculty members create a good overall impression	3.91	0.73	31. Faculty members behave professionally	4.44	0.62
16. I am generally given enough time to understand the things I have to learn	3.47	0.92			
**“Program Design and Delivery” subscale**	3.63	0.27	**“Support & Resources” subscale**	4.19	0.98
32. This program provides a variety of good and relevant courses	3.69	1.03	44. The secretaries are caring and helpful	4.06	0.98
33. The program enhances my analytical skills	3.59	0.95	45. The secretaries behave professionally	4.22	0.75
34. Most courses in this program are beneficial and contribute to my overall professional development	3.56	0.95	46. Support at the clinical and computer labs is readily available	4.13	1.00
35. The quality of instruction I receive in my classes is good and helpful	3.72	0.81	47. Computer and clinical labs are well equipped, adequately staffed, and are readily accessible to meet	4.25	0.67
36. I usually have a clear idea of what is expected of me in this program	3.28	0.89	48. The facilities (class rooms, clinical and computer labs) facilitate my learning	4.31	0.69
37. The program is designed to facilitate team work among students	3.72	0.92			
38. The program enhances my problem solving or critical thinking skills	3.72	0.89			
39. There is a commitment to academic excellence in this program	3.38	1.07			
40. As a result of my courses, I feel confident about dealing with clinical nursing problems	3.09	1.23			
41. Going to class helps me better understand the material	3.88	0.90			
42. I am able to experience intellectual growth in the program	4.06	0.84			
43. Overall, the program requirements are reasonable and achievable	3.88	0.94			
			Total ESAEE Scale	3.75	0.30

**Table 5:** Confirmatory factor analysis (CFA) for ESAEE (rotated component matrix and Cronbach’s alpha coefficient).

**“In-Class Teaching” Subscale (Items 1–16) (Cronbach’s α = 0.94)**	**Factor 1**	**Factor 2**	**Factor 3**	**Factor 4**
3. Faculty members make every effort to assist students when asked	0.729			
14. Faculty members take the time to listen/discuss issues that may impact my academic performance	0.725	0.498		
1. I can freely express my academic and other concerns to faculty members	0.718			
2. Faculty members are easily approachable	0.706			
9. I receive detailed feedback from faculty members on my work and written assignments	0.699			
16. I am generally given enough time to understand the things I have to learn	0.683			
13. Faculty members demonstrate a high level of knowledge in their subject area	0.654			0.442
4. Faculty members make an effort to understand difficulties I might be having with my course work	0.637	0.485		
11. Faculty members are good role models and motivate me to do my best	0.633			
7. Faculty are fair and unbiased in their treatment of individual students	0.632			
8. Faculty members provide adequate feedback about students’ progress in a course	0.624	0.464		
15. Faculty members create a good overall impression	0.617			0.421
12. The administration shows concern for students as individuals	0.604	0.520		
6. I can freely express my academic and other concerns to the administration	0.596			
5. Faculty members are usually available after class and during office hours	0.589			
10. Channels for expressing students’ complaints are readily available	0.588			
**“Clinical Teaching” subscale (items 17–31) (Cronbach’s α = 0.94)**	**Factor 1**	**Factor 2**	**Factor 3**	**Factor 4**
20. Clinical instructors give me sufficient guidance before I perform technical skills		0.881		
28. Clinical instructors provide enough opportunities for independent practice in the lab and clinical sites		0.808		
30. Instructions are consistent among different clinical and lab instructors		0.799		
23. Clinical instructors facilitate my ability to critically assess my client’s needs		0.785		
25. Clinical instructors give me verbal and written feedback concerning my clinical experience		0.784		
18. Clinical instructors provide feedback at appropriate times, and do not embarrass me in front of others (classmates, staff, patients and family members)		0.781		
24. Clinical instructors assign me to patients that are appropriate for my level of competence		0.775		
27. Clinical instructors are available when needed		0.735		
19. Clinical instructors are open to discussions and difference in opinions		0.728		
22. Clinical instructors give me clear ideas of what is expected from me during a clinical rotation		0.718		
29. Clinical instructors encourage me to link theory to practice		0.713		
26. Clinical instructors demonstrate a high level of knowledge and clinical expertise		0.676		
21. Clinical instructors view my mistakes as part of my learning		0.637		
17. Clinical instructors are approachable and make students feel comfortable about asking questions	0.430	0.543		
31. Faculty members behave professionally		0.450		
**“Program Design and Delivery” subscale (items 32–43) (Cronbach’s α = 0.92)**	**Factor 1**	**Factor 2**	**Factor 3**	**Factor 4**
34. Most courses in this program are beneficial and contribute to my overall professional development			0.868	
32. This program provides a variety of good and relevant courses			0.787	
40. As a result of my courses, I feel confident about dealing with clinical nursing problems			0.705	
36. I usually have a clear idea of what is expected of me in this program			0.701	
39. There is a commitment to academic excellence in this program			0.675	
41. Going to class helps me better understand the material			0.677	
38. The program enhances my problem solving or critical thinking skills		0.406	0.650	
35. The quality of instruction I receive in my classes is good and helpful			0.632	
33. The program enhances my analytical skills		0.538	0.613	
42. I am able to experience intellectual growth in the program	0.553		0.671	
37. The program is designed to facilitate team work among students			0.588	
43. Overall, the program requirements are reasonable and achievable	0.471		0.553	
**“Support & Resources” subscale (items 44–48) (Cronbach’s α = 0.92)**	**Factor 1**	**Factor 2**	**Factor 3**	**Factor 4**
46. Support at the clinical and computer labs is readily available				0.875
47. Computer and clinical labs are well equipped, adequately staffed, and are readily accessible to meet				0.760
48. The facilities (class rooms, clinical and computer labs) facilitate my learning				0.746
45. The secretaries behave professionally			0.406	0.648
44. The secretaries are caring and helpful				0.598
Eigen Value				
% of variability	43.835	11.732	8.675	3.070
% of cumulative variability	43.835	55.567	64.242	67.312

Only factor loading values above 0.4 are expressed.

## Supplementary Materials

The following are available online at https://www.mdpi.com/1660-4601/18/2/423/s1, Table S1: “Escala de Satisfacción Académica del Estudiante de Enfermería” (ESAEE scale) after translation, expert committee modifications, and piloting.
