# Supplementary material for: Translation and First Pilot Validation Study of the “Undergraduate Nursing Student Academic Satisfaction Scale” Questionnaire to the Spanish Context
Source: Int J Environ Res Public Health. 2021 Jan 7;18(2):423. doi: 10.3390/ijerph18020423 (PMC7825755; doi:10.3390/ijerph18020423)
Supplement: Supplementary file 1 [file ijerph-18-00423-s001.pdf]

Supplementary Material

Table S1. "Escala de Satisfacción Académica del Estudiante de Enfermería" (ESAEE) scale after translation, expert committee modifications and piloting.

**Ítem**	**Subescala I: Aprendizaje en clase** **Las siguientes preguntas están destinadas a obtener información acerca de tu experiencia de aprendizaje con el plan de estudios del grado**	**Muy de acuerdo**	**De acuerdo**	**Algo de Acuerdo**	**En Desacuerdo**	**Muy en desacuerdo**
1	Puedo expresar con libertad mis inquietudes académicas y de otro tipo a los profesores					
2	Los profesores son fácilmente accesibles					
3	Los profesores se esfuerzan en comprender las dificultades de los estudiantes cuando se les pregunta					
4	Los profesores se esfuerzan por entender las dificultades que podría tener con mi trabajo académico durante la asignatura					
5	Los profesores suelen estar disponibles después de clase y durante las horas de tutoría					
6	Puedo expresar con libertad mis inquietudes académicas y de cualquier otro tipo a la administración					
7	El profesorado es justo e imparcial en su trato con los estudiantes					
8	Los profesores proporcionan comentarios adecuados sobre el progreso de los estudiantes durante la asignatura					
9	Recibo comentarios detallados de los miembros del profesorado sobre mi trabajo y tareas escritas					
10	Los medios para la expresión de las posibles quejas de los estudiantes son fácilmente accesibles					
11	Los profesores son buenos modelos a seguir y me motivan para dar lo mejor de mí					
12	La administración muestra preocupación por los estudiantes como individuos					
13	Los profesores demuestran un alto nivel de conocimiento en su materia					
14	Los profesores se toman tiempo para escuchar/discutir los problemas que puedan afectar en mi rendimiento académico					
15	Los profesores producen una buena impresión general					
16	Generalmente me conceden tiempo suficiente para entender las cosas que debo aprender					
**Ítem**	**Subescala II: Enseñanza clínica** **Las siguientes están destinadas a obtener información acerca de tu experiencia clínica con el plan de estudios del grado**	**Muy de acuerdo**	**De acuerdo**	**Algo de Acuerdo**	**En Desacuerdo**	**Muy en desacuerdo**
17	Los tutores clínicos son cercanos y hacen que los estudiantes se sientan cómodos realizando preguntas					
18	Los tutores clínicos proporcionan comentarios en los momentos apropiados, y no me avergüenzan frente a otros (compañeros de clase, personal, pacientes y familiares)					
19	Los tutores clínicos están abiertos a los debates y la diferencia de opiniones					
20	Los tutores clínicos me proporcionan suficientes					

	orientaciones antes de realizar competencias técnicas					
21	Los tutores clínicos ven mis errores como parte de mi aprendizaje					
22	Los tutores clínicos me dan ideas claras de lo que se espera de mí durante una rotación clínica					
23	Los tutores clínicos facilitan mi capacidad de evaluar críticamente las necesidades del paciente					
24	Los tutores clínicos me asignan pacientes que son apropiados para mi nivel de competencia					
25	Los tutores clínicos me aportan comentarios verbales y escritos en lo referente a mi experiencia clínica					
26	Los tutores clínicos demuestran un alto nivel de conocimiento y experiencia clínica					
27	Los tutores clínicos están disponibles cuando los necesito					
28	Los tutores clínicos brindan suficientes oportunidades para realizar una labor independiente en los talleres de simulación y en las prácticas clínicas					
29	Los tutores clínicos me animan a vincular la teoría con la práctica.					
30	Las instrucciones son coherentes entre diferentes tutores de talleres de simulación y de prácticas clínica					
31	Los profesores se comportan de manera profesional					

**Ítem**	**Subescala III: Diseño y presentación del programa**<br>**Las siguientes preguntas están destinadas a obtener información acerca de tu percepción del plan de estudios del grado y su presentación**	**Muy de acuerdo**	**De acuerdo**	**Algo de Acuerdo**	**En Desacuerdo**	**Muy en desacuerdo**
32	Este plan de estudios ofrece una variedad de asignaturas buenas y relevantes					
33	Este plan de estudios mejora mis competencias analíticas					
34	La mayoría de las asignaturas en este plan de estudio son beneficiosas y contribuyen a mi desarrollo profesional general					
35	La calidad de la instrucción que recibo en mis clases es buena y útil					
36	Por lo general, tengo una idea clara de lo que se espera de mí en este plan de estudios					
37	El plan de estudios está diseñado para facilitar el trabajo en equipo entre los estudiantes					
38	El plan de estudios mejora mis habilidades de resolución de problemas o pensamiento crítico					
39	Hay un compromiso con la excelencia académica en este plan de estudios					
40	Como resultado de mis asignaturas, me siento seguro para tratar con mis problemas clínicos en enfermería					
41	Ir a clase me ayuda a entender mejor el material docente					
42	Cursar este plan de estudios me ha permitido experimentar un crecimiento intelectual					
43	En general, los requisitos del plan de estudios son razonables y alcanzables					

Ítem	**Subescala IV: Apoyo y recursos**<br>**Las siguientes preguntas están destinadas a obtener información acerca de tu percepción del apoyo y los recursos del plan de estudios del grado**	**Muy de acuerdo**	**De acuerdo**	**Algo de Acuerdo**	**En Desacuerdo**	**Muy en desacuerdo**
44	El personal de administración y servicios se interesa por el alumnado y es útil					
45	El personal de administración y servicios se comporta de manera profesional					
46	El material de los talleres de simulación y de las salas de ordenadores está fácilmente disponible.					
47	Los talleres de simulación y las salas de ordenadores están bien equipadas, dotadas de personal adecuado y son fácilmente accesibles					
48	Las instalaciones (aulas, talleres de simulación y salas de informática) facilitan mi aprendizaje					
